# Reduced catabolic protein expression in *Clostridium butyricum* DSM 10702 correlate with reduced 1,3-propanediol synthesis at high glycerol loading

**DOI:** 10.1186/s13568-014-0063-6

**Published:** 2014-08-30

**Authors:** Mine Gungormusler-Yilmaz, Dmitry Shamshurin, Marine Grigoryan, Marcel Taillefer, Victor Spicer, Oleg V Krokhin, Richard Sparling, David B Levin

**Affiliations:** 1 Department of Biosystems Engineering, Faculty of Engineering, University of Manitoba, Manitoba, Winnipeg R3T 5 V6, MB, Canada; 2 Department of Bioengineering, Ege University, Izmir, Turkey; 3 Manitoba Centre for Proteomics and Systems Biology, University of Manitoba, Winnipeg, Manitoba, Canada; 4 Department of Microbiology, University of Manitoba, Winnipeg, Manitoba, Canada; 5 Department of Physics and Astronomy, University of Manitoba, Winnipeg, Manitoba, Canada

**Keywords:** Clostridium butyricum, 1,3-propanediol synthesis, Glycerol catabolism, Proteomics, Multiple reaction monitoring

## Abstract

Higher initial glycerol loadings (620 mM) have a negative effect on growth and 1,3-propanediol (1,3-PDO) synthesis in *Clostridium butyricum* DSM 10702 relative to lower initial glycerol concentrations (170 mM). To help understand metabolic shifts associated with elevated glycerol, protein expression levels were quantified by LC/MS/MS analyses. Thirty one (31) proteins involved in conversion of glycerol to 1,3-PDO and other by-products were analyzed by multiple reaction monitoring (MRM). The analyses revealed that high glycerol concentrations reduced cell growth. The expression levels of most proteins in glycerol catabolism pathways were down-regulated, consistent with the slower growth rates observed. However, at high initial glycerol concentrations, some of the proteins involved in the butyrate synthesis pathways such as a putative ethanol dehydrogenase (CBY_3753) and a 3-hydroxybutyryl-CoA dehydrogenase (CBY_3045) were up-regulated in both exponential and stationary growth phases. Expression levels of proteins (CBY_0500, CBY_0501 and CBY_0502) involved in the reductive pathway of glycerol to 1,3-PDO were consistent with glycerol consumption and product concentrations observed during fermentation at both glycerol concentrations, and the molar yields of 1,3-PDO were similar in both cultures. This is the first report that correlates expression levels of glycerol catabolism enzymes with synthesis of 1,3-PDO in *C. butyricum*. The results revealed that significant differences in the expression of a small subset of proteins were observed between exponential and stationary growth phases at both low and high glycerol concentrations.

## Introduction

1,3-propanediol (1,3-PDO) and terephthalic acid (TPA) are used to produce polytrimethylene terephthalate (PTT), a high performance polymer that is used in apparel, upholstery, carpet, specialty resins and other applications, where properties such as softness, comfort-stretch and recovery, dye ability, and easy-care are desired. Purified 1,3-PDO may also be used in adhesives, resins, laminates, powder coatings, films, solvents (ink-jet dyes), medicines, cosmetics, engine coolants, detergents, insect repellents and fragrances (DuPont [2014]). Demand for PTT was estimated to be approximately 400,000 to 800,000 tones in 2013. Therefore, low-cost production of 1,3-PDO is important for the competitiveness in this market (Nakamura and Whited [2003]).

1,3-PDO may be produced by chemical or biotechnological techniques. Multinational companies, like Degussa (DuPont™) and Shell Chemicals, use chemical processes for 1,3-PDO production (DuPont [2014]; Shell Chemicals [2014]). These processes, however, are high-cost and environmentally hazardous. In contrast, microbial production of 1,3-PDO, for example from glycerol, is less costly and more environmentally sustainable. Although glycerol generated as a by-product of biodiesel production processes has become a “waste” disposal problem for the biodiesel industry, crude biodiesel-derived glycerol can be used as a substrate for microbial production of 1,3-PDO (Clomburg and Gonzalez [2013]). Moon et al. ([2010]) studied the effects of the impurities in biodiesel-derived glycerol and showed that in spite of having inhibitory effects on cell growth, the impurities only slightly affected volumetric yields, suggesting the effective use of biodiesel-derived glycerol for microbial production of 1,3-PDO. However, regardless of its grade, glycerol concentrations over a certain threshold concentration (approximately 600 mM) negatively impact cell growth rates (Zeng et al. [1994]), reduce glycerol consumption, and reduce 1,3-PDO yields (Gungormusler et al. [2011a]). Conversely, greater consumption of glycerol results in greater 1,3-PDO yields. Therefore, it is important to understand the metabolic impacts of glycerol concentrations that negatively effect cell growth and 1,3-PDO synthesis.

Microbial conversion of glycerol to 1,3-PDO and a variety of other fermentation end-products, such as ethanol, acetate, and butyrate, has been well known since the early 1930s (Werkman and Gillen [1932]; Mickelson and Werkman [1940]). Facultative anaerobes such as *Klebsiella pneumoniae*, *Klebsiella oxytoca, Citrobacter freundii* (Pflugmacher and Gottschalk [1994]; Homann et al. [1990]), *Citrobacter werkmanii* (Maervoet et al. [2012]), *Escherichia blattae* (Ripoll et al., [2012]), *Enterobacter aerogenes* and *Enterobacter* (*Pantoea*) *agglomerans* (Ito et al. [2005]; Barbirato et al. [1995]), and strict anaerobes such as *Clostridium beijerinckii* (Gungormusler et al. [2011b]), *Clostridium butyricum*, *Clostridium diolis* (Kaur, et al. [2012]), and *Clostridium pasteurianum* (Biebl [1991]; Biebl et al. [1992]; Gonzalez-Pajuelo et al. [2005]) are the most studied wild-type microorganisms that produce 1,3-PDO. In addition, some species of *Lactobacillus,* such as *Lactobacillus diolivorans* (Pflugl et al. [2012]), and an un-characterized marine species were also reported as possible 1,3-PDO producers when crude glycerol was used as the main substrate (Abad and Turon [2012]). Considering the volumetric productivities and substrate tolerances of the above mentioned bacteria, *C. butyricum* is amongst the best producers (Wilkens et al. [2012]).

In this study, two important factors were taken into consideration for bacteria selection: 1) the tolerance of *C. butyricum* to both high concentrations of 1,3-PDO (up to 93 g/L; Wilkens et al. [2012]) and glycerol (up to 129 g/L, Himmi et al. [1999]); and 2) the non-pathogenic physiology of *C. butyricum*, which does not require special safety precautions to grow, in contrast to *K. pneumoniae* (Saxena et al. [2009]). 1,3-PDO is synthesized predominantly during exponential growth by *C. butyricum* when it is cultured on glycerol as a sole carbon source. During stationary phase, under carbon excess, however, butyrate is synthesized more dominantly by *C. butyricum* (Wilkens et al. [2012]). An understanding of the underlying mechanisms that regulate the formation of fermentation products throughout the phases of microbial growth is essential for development of strategies to maximize 1,3-PDO synthesis.

Proteomics together with end-product analysis can help elucidate possible rate-limiting steps in the metabolic pathways of glycerol degradation to 1,3-PDO. Preliminary studies suggested a growth inhibition after a certain threshold concentration of initial glycerol (620 mM). Hence, the aim of this study was to investigate the expression of key enzymes associated with synthesis of 1,3-PDO and other fermentation end-products (acetate, butyrate, lactate, ethanol, H_2_, and CO_2_) by *C. butyricum* during exponential and stationary phases, under low and high initial glycerol concentrations.

Protein levels were determined using label-free liquid chromatography (LC) based quantitative proteomics using one-dimensional (1D) and two-dimensional (2D) LC/MS/MS, and Multiple Reaction Monitoring (MRM; Walsh et al. [2009]). Our findings suggest a direct correlation between expression levels of key enzymes involved in the glycerol degradation pathways to 1,3-PDO and other end-products. Moreover, our data show that the expression levels of enzymes involved in both the oxidative and reductive pathways of glycerol catabolism are lower at higher glycerol concentrations, with a concomitant reduction in both the glycerol utilisation rate and the cell growth rate.

## Materials and methods

### Organism

*Clostridium butyricum* DSM 10702, which is equivalent to *Clostridium butyricum* 5521 from NCBI, was obtained from the German Type Culture Collection (DSMZ), and was used in all experiments described in this work. The genome sequence used is available through GenBank with the accession no. NZ_ABDT00000000.1.

### Media and growth

The fermentation medium used for the production of 1,3-PDO was as described in Gungormusler et al. ([2011b]). The substrate concentration varied between 4.6 and 85.6 g/L glycerol (50 to 930 mM glycerol) for the trials, whereas for proteomic analysis the media contained 15.6 and 57 g/L (170 mM and 620 mM, respectively) of pure glycerol (initial concentration). Three independent biological replicate cultures were used for each experiment. The triplicate cultures were grown in anaerobic serum bottles (110 mL; Bellco Glass Inc., Vineland, NJ) containing 50 mL of fermentation medium (pH 7.0). All chemicals were obtained from Sigma Chemical Company (St. Loius, MO). Crude glycerol (78-86% w/v glycerol) was obtained from REG Danville, LLC (Danville, IL), and contained major impurities: methanol (0.02% w/v), ash (7% w/v), total fatty acid content (1.0%, w/v) and water (6-13% w/v). All gases were purchased from Welder’s Supplies LTD (Winnipeg, MB, Canada).

*C. butyricum* was activated from lyophilized cultures in fermentation media containing 1 g/L of glucose and incubated anaerobically at 37°C for 24 h. The activated pure strain was used as inoculum (10% v/v) throughout the experiments in 27 mL Balch tubes (with 10 mL working volume) or 110 mL serum bottles (50 mL working volume) with agitation at 100 rpm. The media used for anaerobic experiments were gassed (1 min) with N_2_ gas and degassed (4 min) for 4 cycles before autoclaving (Islam et al. [2006]). Glycerol was used as the sole carbon source for 1,3-PDO synthesis throughout the study. However, revival and growth of *C. butyricum* from glycerol stocks was carried out in rich complex medium containing glucose, as described above.

### Cell growth, pH, and end-product analysis

Cell growth was monitored spectrophotometrically (Biochrom Ultrospec 500 Pro, UK) at 600 nm. End-product analyses were performed as previously described (Gungormusler et al. [2011b]) using high performance liquid chromatography (Waters Breeze™ HPLC System, USA) with a Phenomenex Rezex RHM Monosaccharide (H^+^) 300 x 7.8 mm ion exchange column, using a Waters 2414 Refractive Index Detector, a Waters 1515 Isocratic HPLC Pump, and a Waters 2707 Autosampler. Gas measurements and calculations were carried out as described in Rydzak et al. ([2012]). Data are presented as the means of three independent biological replicates.

### Preparation of peptides for proteomic analysis

Cells grown in medium containing low (170 mM) and high (620 mM) initial glycerol concentrations were harvested for the proteomic analyses described below from exponential and stationary phase cultures. Two biological replicates were used for each 1D and MRM proteomic run, whereas a 2D run was carried out with one sample from the exponential phase of the low initial glycerol concentration cultures. Nine (9) samples in total were analyzed for protein express levels with the various methods, as described below.

All chemicals were sourced from Sigma Chemicals (St-Louis, MO), unless noted otherwise. HPLC-grade acetonitrile and de-ionized water were used for the preparation of eluents. The in-house designed standard peptides (P1-P6) (Krokhin and Spicer [2009]) were custom synthesized by BioSynthesis Inc. (Lewisville, TX) and purified individually using reverse phase (RP) HPLC. Sequencing-grade modified trypsin (Promega, Madison, WI) was used for digestion. A modified “filter-aided sample preparation” (FASP) method (Wisniewski et al. [2009]) was used to extract proteins from *C. butyricum* cells cultured with the two different initial glycerol concentrations, at both exponential and stationary time points. The FASP procedure is based on complete lysis of all cell components using sodium dodecylsulfate (SDS), followed by the subsequent substitution with urea.

Protein amounts to be subjected to digestion were monitored using micro-BCA assay (Thermo Scientific, Pierce, Rockford, IL). Digests were acidified with TFA and purified by RP-HPLC. Protein profiles in the samples were monitored via a SDS-polyacrylamide gel electrophoresis (PAGE) separation. Monitoring the amounts of protein during the digestion procedure is critically important, as a substrate/enzyme ratio of ~50:1 is required for efficient tryptic digestion. Tryptic digests were conducted in the same filter used throughout the experiment containing 1–2 mg/mL protein lysate and 50–100 μg of trypsin. The falcon tubes were replaced with clean 50 mL falcon tubes and incubated overnight in dark at room temperature. Following tryptic digestion, peptide concentrations were estimated using Nanodrop 2000 (Thermo Scientific, Pierce, Rockford, IL). The peptide samples were acidified with TFA to a final concentration of 0.5% and desalted using a 1x100 mm column packed with 5 μm Luna C18 (Phenomenex, Torrance, CA). Purified peptide mixtures were lyophilized and stored at −80°C prior to LC-MS analysis.

Both 1D and 2D LC/MS/MS analyses were employed via identical nano-flow RP-HPLC instrumental settings in the final stage of separation prior to MS acquisition. A splitless nano-flow 2D-Ultra system (Eksigent/ABSciex, Dublin, CA) with 10 μL sample injection via a 300 μm × 5 mm PepMap100 (Thermo Scientific) trap-column and a 100 μm × 200 mm analytical column packed with 5 μm Luna C18 (Phenomenex, Torrance, CA) was used.

### Protein identification and quantitation using LC/MS/MS

Both the 1D and 2D LC/MS/MS analysis were performed using an ABSciex TripleTOF 5600 TOF-MS system (Applied Biosystems, Foster City, CA) equipped with a Nano-sprayIII ionization source. Each acquisition cycle included 250 ms survey MS acqusition (m/z 400–1500) and up to twenty 100 ms MS/MS measurements on the most intense parent ions (300 counts/s threshold, +2 to +4 charge state, m/z 100–1500 mass range for MS/MS). The 1D runs for stationary and exponential phases were performed as biological replicates of two hours runs each.

The resulting raw WIFF files were processed using a standard conversion script bundled with Analyst QS 1.6 into Mascot Generic File format (MGF). MGF files contain information on charge, m/z, retention time of fragmented peptide as well as a list of m/z values and intensities for the CID fragment ions. Mass measurements for the parent and daughter ions are used for peptide identification, while intensities of the daughter ions were employed for quantitation.

All peptide identifications were done with our in-house GPU-based engine (McQueen et al. [2012]). Searches used the 50 most intense fragment peaks with 20 ppm and + −0.1 Da mass tolerances on the parent and fragment masses, respectively. The NCBI annotation of *C. butyricum* was used for peptide identification, with hypothetical tryptic peptides with up to one missed cleavage permitted. The fixed modification of cysteine residues +57.021 Da (cysteine protection with iodoacetamide) was applied to all searches.

The TripleTOF 5600 provides very consistent MS/MS acquisition with excellent linearity between intensity of the parent peptide and its resulting daughter ions. We used protein Total Ion Current (TIC) as a relative measure of protein expression within an experimental run, which is simply the sum of MS/MS fragment ion intensities for every identified member peptide. This value is expressed in a log_2_ scale for simple differential analysis and plotting.

### 1D and 2D LC/MS/MS Analyses

Table 1 shows an overview of protein and peptide identifications for the eight runs. Protein identification confidence expectation values were computed using a Bayes theorem application of its member peptide expectation values, following the design elucidated by Beavis & Fenyo in the X!tandem search engine (http://thegpm.org/docs/peptide_protein_expect.pdf), and is effectively the log_10_ measure of the probability that the protein assignment has a better random answer. Thus an expectation value < −10 has a one-in-10-billion probability of being incorrect.

**Table 1:** An overview of protein and peptide identifications for the 2D and the eight 1D runs

**RUN**	**MS/MS Spectra**	**Total Peptides**	**Non-Redundant Peptides**	**Proteins Log(e) < −3**	**Proteins Log(e) < −10**
**2D-EXP**	301661	133611	17975	2115	1918
**EXP1**	34182	17317	5448	883	722
**EXP2**	35122	17716	5533	902	756
**ST1**	34476	16490	5457	882	728
**ST2**	34526	16088	5298	881	716
**G60EXP1**	28806	13304	5651	980	776
**G60EXP2**	27663	12461	5513	988	788
**G60ST1**	27827	13238	5696	954	768
**G60ST2**	26874	12781	5584	962	764

A generalized analysis system was developed applying a unified data format to quantitation results from a wide range of omics data, across a collection of experiments for a target organism. Values associated with genes/proteins are mapped into Higher Order Variables (HOV) based on broad biological classifications including KEGG modules, COG letters, Enzyme Class numbers and MetaCyc pathways, allowing us to rapidly detect and evaluate system-wide changes in expression levels. These HOV mappings are driven by contents of the “extract gene information” file generated in the IMG/ER annotation workflow (Markowitz et al. [2012]).

The 1D collection of runs was initially filtered to contain only proteins with at least two peptides; this filtering yielded ~ 800 proteins for each growth condition. The expression measurement used for each protein was TIC, measured as the sum of the signal intensities for all peptides derived from a specific protein, and expressed on a log_2_ scale. Differential analysis was constructed by subtracting log2 protein intensity values between points in the growth curve at both glycerol concentrations, and between glycerol concentrations at both points in the growth curve. These were done in replicates, yielding a total of eight differential expression values.

Each set of detected proteins each forms a three-lobed histogram, with the central population of data points containing proteins observed in both states, while the outer histograms contain proteins observed only in one state, but not the other. It is important to emphasize that these are manifestations of the instrument’s information-directed acquisition (IDA) detection limits, and do not represent the absolute presence or absence of a protein in a sample.

For this analysis, we considered only the common populations, containing approximately 500 proteins for each difference set. For each difference set the values were normalized to a mean of zero and standard deviation of one (Z-scores), which exhibit roughly Gaussian distributions: proteins with differences |Z| > 1.65 represent the outermost 10% of the population, and |Z| > 1.96 the outermost 5% of the population. Z-scores for replicates were simply averaged to simplify the analysis, but show reasonable correlations.

Parallels for these eight 1D-IDA proteomic runs were conducted as targeted acquisition via MRM. Since there was precise control of the amount of protein injected for each MRM run, (relatively small) difference means were shifted to 0 only, rather than normalize them to Z-scores. This approach gives fold-change measurements of protein expression levels across the states, which were comparable across the two proteomic methods used.

### Sample preparation and analysis using Multiple Reaction Monitoring (MRM)

Tryptic digests of *C. butyricum* for MRM experiments (QTRAP 5500) were separated using a linear 0.87% acetonitrile per minute gradient (0.4-37.4% acetonitrile in 42.5 min) followed by washing and equilibration steps. Both 1D and 2D analyses were done using the GPU system, with the same search settings.

Stock solutions containing 25 fM/uL of each of six custom peptides and ~1 ug/uL of digests were used for sample preparation by 10-times dilution prior sample injection. 10 uL (IDA) and 20 uL (MRM experiment) sample loop volumes provided injection of ~1 ug of digest (25 fM of standard peptides) and ~2 ug of digest (50 fM of standard peptides) for IDA and MRM runs, respectively.

MRM experiments were performed using QTRAP 5500 (Applied Biosystems, Foster City, CA) hybrid triple quadrupole/ion trap mass spectrometer equipped with a nano-Spray III ion source (2900 V spray voltage and 150°C interface temperature). 60-min MRM methods were used with fixed 10 msec dwell time for each transition and “unit” resolution for both Q1 and Q3 quadrupoles. Collision energy for each transition was calculated according to standard QTRAP 5500 settings.

### Protein identification and quantitation using MRM

The MRM method included 280 transitions from 33 proteins (2–3 peptides per protein, 3–4 transitions per peptide; Table 2; Additional file 1). Retention times of identified peptides were assigned using Skyline. Skyline is a freely available application for building MRM and analyzing the resulting mass spectrometry data (MacLean et al. [2010]).

**Table 2:** The average Z-scores for 1D comparative LC/MS proteomic analyses (four columns after description) and MRM results (last four columns) for the key proteins

**Locus Tag**	**Description**	**ST170 mM- EXP170 mM**	**ST620 mM-EXP620 mM**	**EXP620 mM-EXP170 mM**	**ST620 mM-ST170 mM**	**ST170 mM- EXP170 mM**	**ST620 mM- EXP620 mM**	**EXP620 mM-EXP170 mM**	**ST620 mM-ST170 mM**
**CBY_0205**	phosphate acetyltransferase	−0.04	**1.24**	−0.85	0.25	NQ	0.68	NQ	NQ
**CBY_0206**	acetate kinase	0.00	0.19	−1.00	−0.99	0.25	0.60	−0.73	−0.40
**CBY_0233**	pyruvate:ferredoxin oxidoreductase	−0.84	0.09	0.30	**1.32**	NQ	NQ	NQ	NQ
**CBY_0500**	1.3-propanediol dehydrogenase	−0.26	−0.47	0.06	−0.12	−0.20	−0.32	−0.61	−0.75
**CBY_0501**	glycerol dehydratase (activator)	−0.63	−0.52	**−1.02**	**−1.05**	−0.64	−0.59	**−1.56**	**−1.53**
**CBY_0502**	glycerol dehydratase^a^	−0.34	−0.87	0.24	−0.21	−0.71	−0.74	−0.63	−0.67
**CBY_0505**	dihydroxyacetone kinase. (phosphotransfer subunit)	−0.31	**−1.55**	**−1.14**	**−2.52**	0.18	−0.06	**−1.32**	**−1.57**
**CBY_0506**	dihydroxyacetone kinase (L subunit)	0.47	0.28	0.08	−0.15	0.52	−0.27	−0.15	−0.95
**CBY_0508**	Glycerol dehydrogenase^b^	0.07	−0.19	0.04	−0.24	−0.39	−0.17	**−1.97**	**−1.77**
**CBY_0742**	L-lactate dehydrogenase	0.19	−0.79	**1.08**	0.29	0.22	−0.59	0.26	−0.56
**CBY_1290**	acetyl-CoA acetyltransferase	0.58	0.06	**1.19**	0.83	0.23	0.27	0.68	0.71
**CBY_1889**	pyruvate:ferredoxin oxidoreductase	−0.35	0.00	0.32	0.75	−0.04	0.50	**−1.12**	−0.60
**CBY_2300**	FeFe hydrogenase-1	−0.27	**−1.46**	**1.57**	0.69	−0.27	−0.49	−0.43	−0.67
**CBY_2341**	L-lactate dehydrogenase	0.66	0.10	0.51	−0.02	**1.31**	0.95	0.13	−0.25
**CBY_2757**	L-lactate dehydrogenase	−1.08	−1.43	0.28	0.08	NQ	0.07	NQ	NQ
**CBY_2919**	phosphate butyryltransferase	0.60	0.16	**1.15**	0.86	0.36	0.59	0.00	0.22
**CBY_2920**	butyrate kinase	0.62	0.54	0.89	0.91	0.26	0.32	−0.38	−0.33
**CBY_3041**	3-hydroxybutyryl-CoA dehydratase	0.50	0.29	**1.17**	**1.11**	NQ	NQ	NQ	NQ
**CBY_3042**	acyl-coa dehydrogenase (short-chain specific)	0.20	−0.25	0.90	0.60	−0.06	−0.06	0.96	0.94
**CBY_3045**	3-hydroxybutyryl-coa dehydrogenase	0.24	0.20	**1.16**	**1.28**	0.31	0.06	−0.04	−0.30
**CBY_3235**	Glycerol dehydrogenase^c^	−0.02	−0.52	**−1.47**	**−2.23**	−0.34	−0.36	**−2.66**	**−2.70**
**CBY_3258**	acyl-coa dehydrogenase (short-chain specific)	0.13	0.41	−0.39	−0.20	0.16	0.64	**−1.51**	**−1.05**
**CBY_3642**	pyruvate:ferredoxin oxidoreductase	−0.05	0.29	0.02	0.35	−0.01	0.49	**−1.04**	−0.56
**CBY_3690**	dihydroxyacetone kinase.(L subunit)	0.57	0.75	**−1.75**	**−1.94**	0.13	0.40	**−2.44**	**−2.19**
**CBY_3691**	dihydroxyacetone kinase (DhaK subunit)	0.42	0.58	−0.79	−0.81	NQ	NQ	NQ	NQ
**CBY_3747**	NADPH-dependent butanol dehydrogenase	0.35	−1.12	−0.12	**−1.61**	−0.13	−0.22	**−1.01**	**−1.11**
**CBY_3751**	NADPH-dependent butanol dehydrogenase	0.23	0.08	0.36	0.26	0.53	0.18	−0.57	−0.94
**CBY_3753**	Ethanol dehydrogenase (aldehyde-alcohol dehydrogenase 2)	**−2.58**	**−1.08**	**2.61**	**4.78**	−0.27	−0.12	−0.15	−0.01
**CBY_9999**	glycerol uptake facilitator protein	NQ	NQ	NQ	NQ	NQ	NQ	NQ	NQ

NQ: Not quantifiable. ^a^Wrongly annotated as pyruvate formate-lyase.^b^Wrongly annotated as 3-dehydroquinate synthase.^c^Wrongly annotated as 3-dehydroquinate synthase.

The values indicate the differences between the samples taken from different growth phases (EXP; exponential and ST; stationary) and/or different initial glycerol concentrations (170 mM and 620 mM) of *C. butyricum* cells. Numbers in bold indicate differences in protein measurements that are greater that 2 fold.

**Figure 1:**
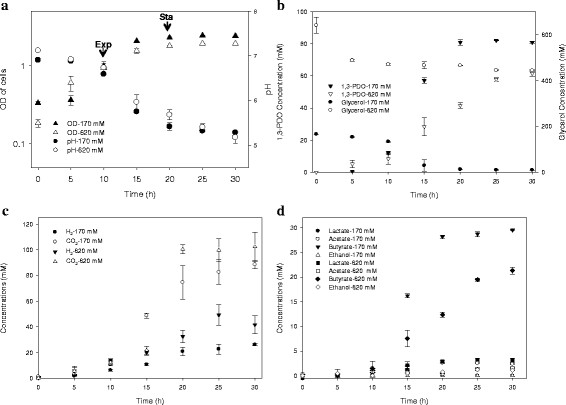
**Growth curves, pH profiles, glycerol utilization, and end-product synthesis profiles of*C. butyricum*cultures with low (170 mM) and high (620 mM) initial glycerol concentrations. a)** OD of cells in log scale versus pH values, **b)** residual glycerol concentrations versus 1,3-PDO concentrations, **c)** H_2_ and CO_2_ concentrations, **d)** lactate, acetate, butyrate and ethanol concentrations.

**Figure 2:**
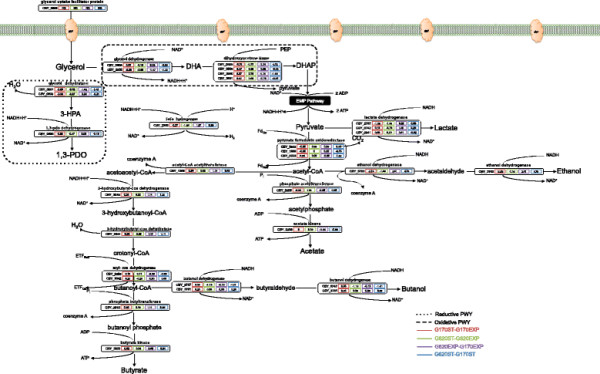
**Differential protein measurements for the proteins in the suggested glycerol catabolism pathways in*C. butyricum*, in average Z-scores for exponential vs. stationary growth phases in media containing low (170 mM) versus high (620 mM) initial glycerol concentrations**. Proteins with significant differences (Z-score > 1 or < −1) in expression between states were shown in bold.

The target enzymes were selected using in-house pathway tools and the NCBI *C. butyricum* 5521 genome sequence. The end-product synthesis patterns were then correlated with the proteomic analyses (Figure 1b, c and d). The enzymes selected for analysis (and their corresponding locus tags) included: glycerol dehydratase (CBY_0501 and 0502), 1,3-PDO dehydrogenase (oxidoreductase) (CBY_0500), glycerol dehydrogenase (CBY_3235 and 0508), dihydroxyacetone kinase (dhaK) (CBY_0505, 0506, 3691 and 3690), pyruvate-ferredoxin oxidoreductase (CBY_0233, CBY_1889 and 3642), FeFe hydrogenase (CBY_2300), acetyl CoA acetyltransferase (CBY_1290), 3-hydroxybutyryl-CoA dehydrogenase (CBY_3045), 3-hydroxybutyryl-coa dehydratase (CBY_3041), acyl-CoA dehydrogenase (CBY_3258 and 3042), phosphate butyryltransferase (CBY_2919), butyrate kinase (CBY_2920), butanol dehydrogenase (CBY_3747 and 3751), phosphate acetyltransferase (CBY_0205), acetate kinase (CBY_0206), lactate dehydrogenase (CBY_0742, 2341 and 2757), and ethanol dehydrogenase (CBY_3753) (Figure 2 and Table 2).

## Results

### Cell growth and end-product analysis

Initial trials to determine the effects of the grade of glycerol on the yields of 1,3-PDO from *C. butyricum* were carried out with 170 mM of initial glycerol (crude versus pure) concentration (data not shown). No significant difference between crude and pure glycerol substrates for the production of 1,3-PDO were observed. Accordingly, further experiments were carried out using pure glycerol. Subsequently, additional experiments were carried out with increasing the initial glycerol concentrations (ranging between 50 mM and 930 mM) in order to understand the threshold values of substrate inhibition (see Additional file 2).

Growth curves of *C. butyricum* cultured with different initial glycerol concentrations revealed a prolonged lag-phase as the initial glycerol concentration increased (see Additional file 2). Cultures with the lowest initial glycerol concentration (50 mM) had a shorter lag phase and entered stationary phase earlier than the cultures with higher initial glycerol concentrations. Cultures with 620 mM initial glycerol concentration displayed longer lag-phases and exponential growth phases, and negligible growth was observed by *C. butyricum* at the two highest initial glycerol concentration cultures (820 and 930 Mm).

1,3-PDO synthesis was observed in cultures with an initial glycerol concentration of up to 820 mM, but the concentrations of 1,3-PDO produced at the highest glycerol concentrations was very low (5 mM; see Additional file 2). Given the negligible growth and synthesis of 1,3-PDO at 820 mM initial glycerol, this concentration was considered to be inhibitory to *C. butyricum*. The highest initial glycerol concentration that was not inhibitory for *C. butyricum* was found to be 620 mM. Acetate, butyrate, lactate, ethanol, H_2_ and CO_2_ were produced in varying amounts during fermentation by most, but not all, cultures with non-inhibitory initial concentrations of glycerol. Butyrate, however, was the only end-product that was observed in all cultures with non-inhibitory initial concentrations of glycerol.

Cell growth curves, pH profiles, and end-product synthesis profiles for *C. butyricum* cultured with two different initial glycerol concentrations are shown in Figure 1. *C. butyricum* uses both reductive and oxidative pathways during the degradation of glycerol (See Discussion section for details). As seen in Figure 1b and 1d, 1,3-PDO was always the primary reduced product followed by butyrate. As both seen in Table 3 and Figure 1b, cultures containing an initial concentration of 620 mM glycerol were always in carbon excess (even after the cells had reached stationary phase, 30 hrs post-inoculation), whereas cultures containing an initial concentration of 170 mM glycerol became carbon limited by 20 hrs post-inoculation, causing growth arrest. However, the molar yields of 1,3-PDO from the two culture conditions were very similar: 0.55 mol 1,3-PDO/mol glycerol consumed for the carbon excess cultures (620 mM initial glycerol) versus 0.51 mol 1,3-PDO/mol glycerol consumed in the carbon limiting cultures (170 mM initial glycerol). Molar yields of butyrate were also similar under both culture conditions, reaching up to 0.22 mol butyrate/mol glycerol consumed in both media.

**Table 3:** ***C. butyricum*** **growth parameters, with aqueous and gaseous end-product concentrations for low (170 mM) vs high (620 mM) initial glycerol concentrations after 20 hours of fermentation**

**Parameter/Glycerol Concentration**	**170 mM**	**620 mM**
C Balance	1.05	1.08
Specific Growth Rate (h^−1^) ^a^	0.175	0.113
Substrate utilization (%)	92.1	28.3
Y PDO/s (mol /mol)	0.51 ± 0.01	0.55 ± 0
PDO (g/L h)	0.31 ± 0.01	0.16 ± 0.01
PDO (g/L)	6.15 ± 0.13	3.13 ± 0.11
Y Buty/s (mol /mol)	0.18 ± 0	0.22 ± 0.01
Buty (g/L)	2.46 ± 0.05	1.28 ± 0.37
Y Acet/s (mol /mol)	0.02 ± 0	0
Acet (g/L)	0.23 ± 0.02	0.04 ± 0
Y H_2_/s (mol /mol)	0.13 ± 0.02	0.19 ± 0.01
H_2_ (mmol/L)	20.84 ± 3.29	32.47 ± 4.76
Y CO_2_/s (mol /mol)	0.47 ± 0.08	0.60 ± 0.01
CO_2_ (mmol/L)	74.76 ± 12.84	100.58 ± 3.42

^a^Growth rate was calculated from the increase in number of cells with time during the exponential phase. Carbon recovery was calculated from the ratio of (carbon out)/(carbon in) where the formula C_4_H_7_O_2_N (Zeng et al., [1993]) was used for *C. butyricum* biomass; Y, Yield; PDO, 1,3-PDO; Buty, butyrate; Acet, acetate. Three independent biological replicates were used for each experiment.

As stated in Table 3, the specific growth rate, was lower in the high glycerol concentration cultures, suggesting growth inhibition. Although high glycerol concentrations reduced growth of *C. butyricum*, they did not significantly affect the substrate-specific yield of 1,3-PDO (mol 1,3-PDO per mole glycerol consumed). A previous study with *C. butyricum* also reported decrease in the growth rate as the total osmolarity of the medium increased (Biebl [1991]), which may also be observed in 620 mM glycerol.

### Protein identification in the core metabolic pathways

One 2D LC/MS/MS run (20 x 1 h runs) (Dwivedi et al., [2008]) was conducted on an exponential phase sample from cells grown in low glycerol (170 mM) (see Additional file 1), primarily to survey the microorganism’s proteome for future cross-organism and RNAseq studies. Detailed discussion of these results are outside the scope of this paper, but it does help validate the 1D-quant approach: while this instrument run set gave almost 9 times as many MS/MS spectra as the 1D runs, the number of highly confident proteins (log(e) < −10) identified increased only by a factor of 2.6 (~1900 proteins). This supports the notion that this genome is sufficiently simple (<4000 genes) to apply 1D quantitation analysis for accessing biologically relevant processes, especially catabolic processes.

Changes in the proteome during the exponential and stationary phases of *C. butyricum* cells, cultured in low and high initial glycerol concentrations, were further investigated using 1D label-free LC/MS/MS proteomics. A total of 3071 genes were annotated in the *C. butyricum* genome (Table 1) with total TIC scores spanning from ~ 12 to 27 units (in log_2_ scale; see Additional file 1) for 170 mM glycerol exponential phase, 170 mM glycerol stationary phase, 620 mM glycerol exponential phase, and 620 mM glycerol stationary phase, respectively.

The first objective of this work was to elucidate the key enzymes involved in the conversion of glycerol to 1,3-PDO and other fermentation end-products by 1D LC/MS/MS analysis on the basis of observed TIC scores. The second objective was to compare the expression levels of the targeted proteins using MRM, which is one of the most sensitive methods for quantification of known analytes (Kitteringham et al. [2009]). All the enzymes of interest were expressed at detectable levels under all the conditions studied (see Additional file 1).

The intra-sample replicates and the cross sample replicates for both 1D and MRM analysis showed a linearity that demonstrates the similarity of the data, indicating that the data for 1D and MRM are highly correlated. The proteins selected for MRM were mainly found around the centroid (see Additional file 2) and below the centroid (see Additional file 2) when cross comparisons between glycerol concentrations and/or between growth phases were used, respectively. These results demonstrated that the selected proteins did not change in the same way in response to glycerol concentrations and growth phases. Specifically (see Additional file 2), the data generated from the intra-treatment replicate sample pairs for the two different initial glycerol concentrations were highly similar for both stationary and exponential phase samples. On the basis of these results and similar correlation trends, average values of the differences between states were used (Table 2). In addition, comparison of data derived from MRM analyses of exponential versus stationary phases, resulted in data that clustered more towards to the middle of the overall data set, indicating little change in the overall protein complement of core catabolic proteins examined, as the cells transitioned from exponential to stationary phase. Whereas comparison of data derived from MRM analyses of samples from low versus high initial glycerol concentrations resulted in data that were more spread-out across the overall data set (see Additional file 2).

Significant differences in the expression levels of the key proteins involved in glycerol catabolism, 1,3-PDO synthesis (CBY_0500, 0501 and 0502) were observed in cultures with different initial glycerol concentrations (Table 2). Specifically, a decrease was detected in CBY_0501 in higher glycerol concentration. Likewise, proteins corresponding to CBY_0505, 3235, 3690 and 3691 associated with the energy yielding oxidative arm of glycerol metabolism were also reduced in significant amounts in the presence of high glycerol. The TIC scores of the genes CBY_0506 and 3691 related to the synthesis of putative dihydroxyacetone kinase only slightly decreased in amounts during the stationary phase of growth in high glycerol relative to the low glycerol concentration in the stationary phase of growth (see Additional file 1).

In general, enzymes involved in butyrate synthesis were detected in higher amounts for both growth phases in high glycerol cultures, except for acyl-CoA dehydrogenase (CBY_3258), which showed lower expression levels in both exponential and stationary growth phases in high initial glycerol concentration cultures. Phosphate acetyltransferase and acetate kinase (CBY_0205 and 0206) also showed lower amounts in high glycerol except for the stationary phase in CBY_0205 (Table 2).

Transportation of glycerol through the cell membrane can be carried out by (GlpF), which forms a highly selective trans-membrane channel that conducts glycerol and small, uncharged organic molecules (Stroud et al. [2003]), thus maintaining the membrane potential. A putative glycerol uptake facilitator, CBY_9999, was detected in the proteome of *C. butyricum* (see Additional file 1) according to the 1D-IDA analysis. Although these findings cannot be supported by MRM results, it is important to point out that the expression levels (as measured by the TIC scores) of this protein were above detectable levels. However, when the initial glycerol concentration of the culture was altered, only slight changes in the expression of CBY_9999 were observed.

According to the proteome analyses, expression levels of most *C. butyricum* enzymes did not change significantly under the two growth conditions tested (Table 2). However, a key subset of proteins involved in the synthesis of 1,3-PDO from glycerol did display significant differences, including enzymes involved in glycerol oxidation, glycerol reduction to 1,3-PDO production, and butyrate synthesis were up or down regulated in the high glycerol concentration cultures as discussed above.

## Discussion

### Glycerol uptake

Glycerol is an osmolyte, and changes observed in cells grown in media with different initial glycerol concentrations may be explained by possible changes in osmotic pressure in the cell (Kubiak et al., [2012]). Several studies have reported on the effect of various glycerol concentrations (Zhang et al. [2007]; Sattayasamitsathit et al. [2011]; Metsoviti et al. [2012]; Kang et al. [2013]). High initial glycerol concentrations have been observed to result in lower glycerol consumption rates, with prolonged fermentation times. In addition, depending on the species and operational conditions, certain threshold concentrations of glycerol (e.g. >60 g/L for *C. butyricum*, as reported in this study) inhibit bacterial growth.

A study by Sattayasamitsathit et al. ([2011]) showed that as the initial glycerol concentration increased above 60 g/L (up to 100 g/L), *Klebsiella pneumoniae* SU6 cells started to consume less glycerol, indicating a possible growth inhibition as a result of the higher concentration of certain impurities in the crude glycerol. *Citrobacter freundii* produced lower concentrations of 1,3-PDO (45.9 g/L) as the initial glycerol concentration increased to 100 g/L, suggesting growth inhibition (Metsoviti et al. [2012]). A decrease in 1,3-PDO production (26%) with a concomitant increase in ethanol production (15%) was reported by *Lactobacillus panis* when the initial glycerol concentration was doubled from 150 mmol/L to 300 mmol/L (Kang et al. [2013]). The authors suggested that glycerol fermentation was in competition with ethanol production and thus served as an alternate route for NAD regeneration. Our findings with *C. butyricum* are consistent with these results, suggesting an alteration in the regeneration routes of NADH as the initial glycerol concentration increases. In contrast to *L. panis,* which synthesized more ethanol as the initial glycerol concentration increased, we observed an increase in lactate synthesis by *C. butyricum.*

Glycerol can be transported through the membrane either by passive diffusion or via the glycerol facilitator protein (a glycerol permease), GlpF (da Silva et al. [2009]; Sun et al. [2008]). Therefore, the transportation through the membrane is done by diffusion and ATP utilization is not required. The results from the proteomic analysis (1D-IDA only) showed that GlpF (CBY_9999; Figure 2) was detected in cells grown in both low and high initial glycerol concentrations (except for one biological replicate in the exponential phase of the high glycerol concentration cultures). However, the TIC scores for this protein, were near the lower detection limit when detected, indicating that the facilitator protein may not play a major role at either of the glycerol concentrations tested.

### Reductive pathways of glycerol degradation

The yields and productivities that can be achieved from the degradation of a specific substrate are adjusted such that ATP gain and thermodynamic efficiency are optimal for the respective growth conditions. Although the thermodynamic efficiency of ATP synthesis through acetate production pathways was reported to be higher in *C. pasteurianum* (85%) than the efficiency of acetate production pathways (63%), butyrate was always produced suggesting an incompatibility of high efficiency with the entropy requirements of the cells (Thauer et al. [1977]). In addition, reducing equivalents are formed simultaneously in the form of reduced NADH during the formation of biomass (via the oxidation of glycerol), since glycerol has a higher degree of reduction per carbon than the cell mass (CH_1.9_O_0.5_ N_0.2_). In this case, the ability of the microorganism to maintain an overall redox balance would also be relevant to its ability to produce a product more reduced than glycerol by the consumption of the reducing equivalents (Nakamura and Whited [2003]). When compared with sugars, such as glucose, this degradation to pyruvate generates twice the number of reducing equivalents. Regeneration of oxidized nicotinamide adenine dinucleotide (NAD^+^) requires the formation of a more reduced end-product, such as 1,3-PDO, to serve as an electron sink (Nakamura and Whited [2003]; Clomburg and Gonzalez [2013]).

*C. butyricum* is a strict anaerobe, and maintains a balanced redox by utilizing two pathways (Figure 2). In the reductive pathway, glycerol is first dehydrated to the toxic intermediate 3-hydroxypropionaldehyde (3-HPA) and then 3-HPA is reduced to 1,3-PDO by the regeneration of oxidized NAD^+^ using 1,3-PDO dehydrogenase. A study to increase the NADH availability in *K. pneumoniae* was carried-out by Ma et al. ([2012]) with the introduction of an *fdh* from *Candida boidinii* into the microorganism. The results were in agreement with our statement and demonstrated that the increase of NADH availability could efficiently improve glycerol metabolism, suggesting that increased 1,3-PDO yields are not only controlled by expression levels of key enzymes, but also by the intracellular concentration of NADH.

On the basis of the results (Figure 2), the total flux of carbon through both the glycerol reductive and oxidative branches are reduced in the presence of 620 mM glycerol. This is consistent with the proteomic results which show greater than 2-fold reduction in expression for key enzymes from both branches: putative glycerol dehydratase (CBY_0501), glycerol dehydrogenase (CBY_3235), and DhaK (CBY_0505 and 3690), which are key enzyme components in the reductive and oxidative pathways affected by high glycerol concentrations. Interestingly, many of these are located in the same genome neighbourhood (except for CBY_3691, 3690 and 3235), possibly forming a transcriptional operon. This putative operon would manage both reductive and oxidative branches, leading to balanced pathway utilization.

Abbad-Andaloussi et al. ([1996]) reported that glycerol dehydratase prevents intracellular accumulation of 3-HPA, therefore it is important to have a high glycerol dehydratase activity in order to increase the 1,3-PDO yields. According to the authors, based on excess NADH measurements, glycerol dehydratase limits the activity of 1,3-PDO dehydrogenase and it is the rate-limiting step in this fermentation. In this paper, glycerol dehydratase levels (CBY_0502) were also always higher than 1,3-PDO dehydrogenase levels (CBY_0500) based on their TIC scores (see Additional file 1). Gonzalez et al. ([2013]) used 2D gel electrophoresis coupled to MS analysis to compare the differentially expressed proteins in *Clostridium* sp. native strain (IBUN 158B) in two phases of growth (exponential and stationary) during 1,3-PDO synthesis. Thirty-two proteins were identified in the Gonzalez et al. ([2013]) study, including 1,3-PDO dehydrogenase, which was detected in both exponential and stationary phases. Our results are consistent with these results.

A recent study by Zhu et al. ([2014]) reported the effects of altered oxido-reduction potential (ORP) in protein expression by combined proteomic profiling and flux balance analysis in *K. oxytoca* cultures. The authors stated that the ratio of metabolic fluxes through reductive and oxidative pathways of glycerol degradation were sensitive to changes in extracellular ORP, and reductive pathways were channelled by low ORP. In addition, 2D gel electrophoresis showed that low ORP correlated with increased expression levels of an NADPH-dependent hypothetical oxidoreductase (HOR), but did not alter expression of 1,3-propanediol oxidoreductase. In our study, although the TIC values of 1,3-propanediol oxidoreductase (as also known as 1,3-propanediol dehydrogenase) were significantly high, suggesting the importance of this enzyme in the production of 1,3-PDO, the expression levels of the enzyme only slightly changed in both conditions studied.

### Oxidative pathways of glycerol degradation

In the oxidative pathway, glycerol is first oxidized to dihydroxyacetone (DHA) (also known as glycerone) using NAD^+^ as a cofactor by glycerol dehydrogenase. Then DHA is phosphorylated to dihydroxyacetonephosphate (DHAP) using a phosphoenolpyruvate dependent DHA kinase. In some clostridia glycerol can also be utilized via a glycerol kinase and glycerol-P-dehydrogenase pathway (Gonzalez-Pajuelo et al. [2006]). Interestingly, a putative glycerol-P-dehydrogenase was detected in relatively high abundance in the proteome of *C. butyricum*, but it is not believed to utilize this pathway for glycerol oxidation as the TIC scores of the putative glycerol kinase was below detectable levels (see Additional file 1). Wang et al. ([2003]) compared the differences in growth phases in fed-batch fermentation by *K. pneumoniae* using 2D gel electrophoresis. The authors stated that this strain also uses a PEP-dependent DhaK as opposed to the ATP-dependent enzyme previously reported for *K. pneumoniae* and *C. freundii*.

Following DHAP production, it enters the Embden-Meyerhof-Parnas (EMP) pathway for glycolysis, resulting in a net gain of 2 moles of ATP and 1 mole of NADH per mole of DHAP. Following the EMP pathway, pyruvate can be reduced to lactate or oxidized to acetyl-CoA plus CO_2_. Electrons from this oxidation can be used for hydrogen production allowing the cells to conserve further energy (ATP) through the conversion of acetyl-CoA to acetate through acetyl-CoA phosphotransferase and acetate kinase. In the presence of hydrogen, there are excess reducing equivalents in the pool that can further be converted into other reduced compounds in order to maintain the redox balance.

Initially, the conversion of pyruvate to acetyl-coA by the utilization of pyruvate-ferrodoxin oxidoreductase results in the formation of CO_2_ and a reduced ferredoxin (Figure 2). This reduced ferredoxin is then used as an electron transporter for the formation of butanoyl-CoA by acyl-CoA dehydrogenase, which is then further converted to butyrate following two more steps. As a result, a ratio of 1 CO_2_ to 1/2 butyrate (mol:mol) is maintained following the degradation of pyruvate. Butyrate production leads to 0.5 mole of ATP produced whereas acetate production leads to 1 mole of ATP produced for every pyruvate consumed. Considering that acetate production does not consume NADH as opposed to butyrate, this pathway favours 1,3-PDO production. However, under the experimental conditions, in the presence of hydrogen in the atmosphere, butyrate was produced at significantly higher levels than acetate (Figure 1d). Our data are consistent with the 2D gel electrophoresis-MS proteomic study of Gonzalez et al. ([2013]), which showed that the enzymes in the butyrate synthesis pathways (CBY_3041, 3045, 3258, 3042, 2919, and 2920) were present in both exponential and stationary growth phases.

All the by-products of glycerol catabolism (lactate, ethanol, butanol, H_2_, and particularly butyrate) compete with 1,3-PDO for reducing equivalents (NADH) during their synthesis. While lactate production from pyruvate uses 1 mole of NADH, ethanol production from acetyl-CoA through acetaldehyde results in the consumption of 2 moles of NADH. In addition, ethanol synthesis results in the formation of CO_2_ and a reduced ferredoxin. As seen in Figure 2, pyruvate catabolism to acetyl-CoA generates a reduced ferredoxin that can be re-oxidized in the formation of H_2_ or possibly for butyrate synthesis (ETF_Red_ to ETF_Ox_). Although a putative hydrogenase (CBY_2676) was annotated in the genome of *C. butyricum*, in our study, it was below detectable levels in the proteome. Thus, butyrate might be the strongest competitor for NADH.

Theoretically, while acetic acid formation leads to the formation of 1 mole of excess NADH per mole of acetic acid formed, butyrate and lactate synthesis do not result in excess NADH per mole of the end-product formed, since NADH is utilized by the dehydrogenases (Kubiak et al. [2012]). The excess NADH might be utilized if butanol or ethanol pathways are active for the conversion of acetyl-CoA into these solvents. The ratio of acetate to butyrate can be related to the metabolic status of the cell, and the increase in the acetate:butyrate ratio (acetate concentration/butyrate concentration) may act as an indicator of a faster microbial growth. Our results were consistent with this statement. As seen in Table 3, growth of *C. butyricum* was faster in low initial glycerol concentration cultures. Accordingly, the acetate:butyrate ratio was higher in low initial glycerol concentration cultures (1.17) than high initial glycerol concentration cultures (0.25).

The effects of various end-products synthesized during the oxidative degradation of glycerol on *C. butyricum* growth has been analyzed in some detail (Colin et al. [2000]; Barbirato et al. [1996], Biebl [1991]). (Colin et al. [2000]) showed that *C. butyricum* cells had better tolerance to higher concentrations of internally produced 1,3-PDO (83.7 g/L) than externally added 1,3-PDO (65 g/L). Accordingly, the authors related the inhibitory effects of the diols (1,3-PDO, 1,2-PDO, 2,3-BD) to the general phenomenon of alcohol inhibition in microbial fermentations. Another study by Biebl ([1991]) reported the concentrations of acetate (27 g/L), butyrate (19 g/L), and 1,3-PDO (60 g/L) that were inhibitory to *C. butyricum* and showed that 1,3-PDO was the main inhibitor. However, the acetate:butyrate ratio was dependent on the environmental conditions, such as pH and glycerol concentration, and could reduce the production of 1,3-PDO. Chatzifragkou et al. ([2012]) compared the effects of high 1,3-PDO concentrations (biologically produced versus externally added, up to 70 g/L) in batch and continuous cultures. The results showed that *C. butyricum* was less resistant to the inhibitory effects of diols in batch cultures than in continuous cultures.

Although neither butanol nor ethanol were detected (Figure 1d), the *C. butyricum* genome contains the putative genes that encode the enzymes required for synthesis of these end-products during glycerol fermentation (Saint-Amans et al. [2001]). In addition, the proteomic analyses revealed that the enzymes involved in ethanol synthesis were expressed and were detected at levels above detectable limits (TIC scores around 22.6, see Additional file 1). However, end-product analysis revealed the fact that expression of a protein does not necessarily correlate with the formation of the expected end-product, most likely because of post-translational regulation such as allostery (Monod et al. [1963]). Similarity, despite the lack of butanol production, the enzymes responsible for butanol synthesis were also expressed in the proteome. As seen in Table 2, one putative alcohol dehydrogenase (CBY_3747) was down-regulated, while another putative alcohol dehydrogenase (CBY_3751) was up-regulated, in the higher initial glycerol concentration cultures (Figure 1c). The putative ethanol synthesizing alcohol dehydrogenase, AdhE (encoded by CBY_3753), was expressed at higher levels during both exponential and stationary phase in high initial glycerol concentration cultures than in low glycerol concentration cultures. Initial trials to determine the threshold for ethanol consumption by *C. butyricum* showed that the microorganism can consume ethanol up to a concentration of 25 mM (data not shown) and this could possibly explain the lack of net ethanol production.

A second possible explanation this observation could be the competition for NADH. A mutant strain of *C. butyricum* (W5) with a blocked butyrate synthesis pathway revealed that during glucose fermentation, increased hydrogen production leads to decreased ethanol formation, even though the opposed was expected. The authors suggested that a bifurcating hydrogenase encoded by *C. butyricum* W5 may compete for NADH (Cai et al. [2011]).

In addition to a lack of ethanol, there was also no evidence for lactate production in our low glycerol concentration cultures. However, up to 2.9 mM of lactate was produced when initial glycerol concentration was increased (Figure 1d). This could be explained by the higher expression levels of two of the genes encoding LDHs (CBY_0742 and CBY_2341) during exponential phase in the high glycerol concentration cultures, based on their TIC scores. On the other hand, for all possible genes encoding LDHs (CBY_2757, CBY_0742, and CBY_2341), the expression levels did not change significantly during stationary phase in the high glycerol concentration cultures.

In *C. butyricum*, under excess substrate, NADP^+^:ferredoxin oxidoreductase (CBY_3749) can be utilised to transfer the electrons from ferredoxin to NAD^+^ instead of being released as H_2_, contributing to additional 1,3-PDO formation (Leja et al. [2011]). Our proteomic analysis, however, showed the presence of an annotated single sub-unit, ferredoxin-dependent, iron-only hydrogenase (CBY_2300) in relatively high abundance (see Additional file 1), which suggests that the observed H_2_ production in *C. butyricum* can be carried out by the oxidation of reduced ferredoxin derived from the oxidation of pyruvate to acetyl-CoA and CO_2_ through pyruvate ferredoxin oxidoreductase (CBY_0233, 1889 and 3642). A hydrogenase can also act as a safety valve to maintain the redox balance by using the excess reducing equivalents due to the fact that this is an exergonic reaction and no energy is conserved (Madigan et al. [2009]).

In the proteome of *C. butyricum*, when the initial glycerol concentration of glycerol was increased, most of the pathways for the synthesis of soluble end-products (1,3-PDO, lactate, butanol, butyrate, acetate and ethanol), except for butyrate, lactate and ethanol (CBY_3045, 0742 and 3753), were down-regulated in both growth phases (Table 2). Furthermore, the key proteins that control flux of glycerol through the reductive and oxidative branches were down-regulated which is consistent with the lower 1,3-PDO concentrations in high glycerol concentration cultures (Figure 1b). In addition, based on the TIC scores, the putative ethanol dehydrogenase was expressed in considerable amounts in the proteome (CBY_3753, see Additional file 1), even though there were low concentrations of ethanol in the cultures with high initial glycerol concentration and no detectable production of ethanol was observed in cultures with low initial glycerol concentration.

The low concentration of ethanol in the high glycerol cultures could be attributed to the lower rate of glycerol consumption and lower growth rate in the presence of high glycerol concentration (Table 3). The expression levels of enzymes in a recombinant *E. coli* engineered to synthesize 1,3-PDO were analyzed by 2D gel electrophoresis. The results showed that as the initial glycerol concentration of the cultures was reduced, the major enzymes, expression of all increased and synthesis of 1,3-PDO was enhanced (Jin and Lee [2008]). Our observations were consistent with these findings.

This paper correlates expression levels of key enzymes involved in glycerol catabolism and 1,3-PDO synthesis with end-product synthesis rates. This is the first study reporting the changes in the proteomic profiles of *C. butyricum* cells under different culture conditions using comparative proteomic analysis. The results of this study showed that higher glycerol concentrations can reduce the expression levels of enzymes involved in both pathways (oxidative and reductive), consistent with lower growth rate and glycerol utilisation rate. The results indicate that there is an optimum initial loading of glycerol for fastest growth rate and 1,3-PDO production. However, there is stability in the proportion of 1,3-PDO produced relative to other end-products generated under the growth conditions and growth phases tested. Nevertheless, the proteomic results described can serve as baseline for optimization of fermentation or selection of targets for genetic engineering for end-product optimization which could lead to more efficient production processes with low cost downstream processes and higher final yields of the main product.

## Competing interests

The authors declare that they have no competing interests.

## Additional files

## Supplementary Material

Additional file 1 **Data from 2D, 1D LC/MS/MS, and MRM proteomic analyses.** Expression levels for each protein are indicated as the Total Ion Current (TIC), measured as the sum of the signal intensities for all peptides associated with each specific protein, and expressed on a log2 scale. Click here for file

Additional file 2 **Growth curves and end-product synthesis profiles of *C. butyricum* cultures with increasing (50, 170, 300, 620, 820, 930 mM) initial glycerol concentrations.** a) Growth curves, b) 1,3-PDO, butyrate, acetate, and lactate concentrations, c) H_2_ and CO_2_ concentrations. Correlation between the Z-scores of all the proteomics data between intra- and cross-replicate samples for both 1D-IDA (closed circles) and MRM (open circles) analyses. Differences between a) 170 and 170 mM, b) 620 and 620 mM, c) 170 and 620 mM, d) 170 and 620 mM initial glycerol concentrations. ST, Stationary phase; EXP, Exponential phase; R1, Replicate one; R2, Replicate two. Correlation between the Z-scores of intra- and cross-replicate samples: a) 1D, b) MRM, c) 1D versus MRM analysis for key proteins in exponential versus stationary growth phase, in cultures with low (170 mM) versus high (620 mM) initial glycerol concentrations. ST, Stationary phase; EXP, Exponential phase; R1, Replicate one; R2, Replicate two. Click here for file
