# Supplementary material for: Reduced catabolic protein expression in Clostridium butyricum DSM 10702 correlate with reduced 1,3-propanediol synthesis at high glycerol loading
Source: AMB Express. 2014 Aug 30;4:63. doi: 10.1186/s13568-014-0063-6 (PMC4230902; doi:10.1186/s13568-014-0063-6)
Supplement: Additional file 2 — Growth curves and end-product synthesis profiles of C. butyricum cultures with increasing (50, 170, 300, 620, 820, 930 mM) initial glycerol concentrations. a) Growth curves, b) 1,3-PDO, butyrate, acetate, and lactate concentrations, c) H2 and CO2 concentrations. Correlation between the Z-scores of all the proteomics data between intra- and cross-replicate samples for both 1D-IDA (closed circles) and MRM (open circles) analyses. Differences between a) 170 and 170 mM, b) 620 and 620 mM, c) 170 and 620 mM, d) 170 and 620 mM initial glycerol concentrations. ST, Stationary phase; EXP, Exponential phase; R1, Replicate one; R2, Replicate two. Correlation between the Z-scores of intra- and cross-replicate samples: a) 1D, b) MRM, c) 1D versus MRM analysis for key proteins in exponential versus stationary growth phase, in cultures with low (170 mM) versus high (620 mM) initial glycerol concentrations. ST, Stationary phase; EXP, Exponential phase; R1, Replicate one; R2, Replicate two. [file s13568-014-0063-6-S2.doc]

**a) b)** **c)**

Online Resource 2. Growth curves and end-product synthesis profiles of *C. butyricum* cultures with increasing (50, 170, 300, 620, 820, 930 mM) initial glycerol concentrations. a) Growth curves, b) 1,3-PDO, butyrate, acetate, and lactate concentrations, c) H_2_ and CO_2_ concentrations.

**a****bcd**

Online Resource 3. Correlation between the Z-scores of all the proteomics data between intra- and cross-replicate samples for both 1D-IDA (closed circles) and MRM (open circles) analyses. Differences between a) 170 and 170 mM, b) 620 and 620 mM, c) 170 and 620 mM, d) 170 and 620 mM initial glycerol concentrations. ST, Stationary phase; EXP, Exponential phase; R1, Replicate one; R2, Replicate two.

**a**

**b**

**c**

Online Resource 4. Correlation between the Z-scores of intra- and cross-replicate samples: a) 1D, b) MRM, c) 1D versus MRM analysis for key proteins in exponential versus stationary growth phase, in cultures with low (170 mM) versus high (620 mM) initial glycerol concentrations. ST, Stationary phase; EXP, Exponential phase; R1, Replicate one; R2, Replicate two.

**Reduced catabolic protein expression in *Clostridium butyricum* DSM 10702 correlate with reduced 1,3-propanediol synthesis at high glycerol loading**

Applied Microbiology and Biotechnology Express

Mine Gungormusler-Yilmaz, Dmitry Shamshurin, Marine Grigoryan, Marcel Taillefer, Victor Spicer, Oleg V. Krokhin, Richard Sparling, and David B. Levin^*^

* Corresponding author. David B. Levin, University of Manitoba, Department of Biosystems Engineering, E-mail address: [david.levin@umanitoba.ca](mailto:david.levin@umanitoba.ca)
